# Pesticide Residues in Fruits: From Surveillance Data to Risk-Based Interpretation and Mitigation

**DOI:** 10.3390/molecules31111980

**Published:** 2026-06-05

**Authors:** Jarosław Chmielewski, Barbara Gworek, Ewa Beata Górska, Maciej Masłyk, Łukasz Szarpak, Grażyna Nowak-Starz

**Affiliations:** 1 Institute of Environmental Protection-National Research Institute, 02-170 Warsaw, Poland; barbara.gworek@ios.edu.pl; 2 Department of Biochemistry and Microbiology, Institute of Biology, Warsaw University of Life Sciences, Nowoursynowska St. 166, 02-787 Warsaw, Poland; ewa_gorska@sggw.edu.pl; 3 Institute of Biological Sciences, The John Paul II Catholic University of Lublin, 20-950 Lublin, Poland; maciej.maslyk@kul.pl; 4 Institute of Medical Sciences, The John Paul II Catholic University of Lublin, 20-950 Lublin, Poland; lukasz.szarpak@kul.pl; 5 Collegium Medicum, Jan Kochanowski University, 25-369 Kielce, Poland; gnowakstarz@wp.pl

**Keywords:** pesticide residues, fruits, cumulative risk assessment, maximum residue levels, transformation products

## Abstract

**Background:** Interpretation of pesticide residues in fruits requires tight integration of surveillance evidence, analytical capability, regulatory context, and mitigation data. **Methods:** This critical integrative review synthesises analytical chemistry, cumulative risk assessment (CRA), regulatory divergence, and mitigation evidence, strengthened by quantitative monitoring summaries and auditable regulatory examples. Routine enforcement continues to rely on validated QuEChERS extraction coupled with targeted LC-MS/MS and GC-MS/MS. High-resolution mass spectrometry (HRMS) adds unique value for metabolites, transformation products (TPs), and incident response, but its routine enforcement role remains constrained by confirmation logic and harmonised validation. **Results:** Monitoring shows that exposure is typically multi-residue rather than single-compound; the key interpretive challenge therefore shifts toward CRA prioritisation, sensitive-subpopulation assumptions, and transparent distinction between compliance signals and toxicological inference. We provide (i) headline compliance metrics from EU and US programmes, (ii) surveillance-derived high-frequency residue patterns and co-occurrence motifs to guide CRA prioritisation, (iii) an illustrative, traceable comparison of EU/US/Codex MRL divergence for emblematic citrus residues with EU evidence extracts and US/Codex traceability records, and (iv) mitigation evidence statements standardised by study type and transformation-product reporting. **Conclusions:** Pesticide residues in fruits should be interpreted through a risk-based framework that distinguishes compliance findings from toxicological concern, prioritises relevant multi-residue drivers, and evaluates mitigation according to both residue reduction and transformation-product uncertainty.

## 1. Introduction

Pesticide residues in fruit should be interpreted within food-safety and food-toxicology frameworks, because these domains define surveillance priorities, analytical strategies, and interpretation of residue findings. Within food-systems research, the essential scientific task is not simply to catalogue detections, but to distinguish rigorously between compliance monitoring, exposure assessment, and toxicological interpretation. Treating these distinct domains as a single, undifferentiated narrative of consumer risk weakens both regulatory clarity and scientific precision. In regulated markets, meaningful interpretation therefore depends on the integration of occurrence data, analytical performance characteristics, mixture-aware risk concepts, and evidence addressing residue reduction in realistic fruit matrices.

Two parallel realities define the current landscape. First, surveillance data from regulated markets, including programmes coordinated by the European Food Safety Authority (EFSA), consistently demonstrate high compliance with established standards [1,2,3]. In 2022, 96.3% of samples analysed in the European Union (EU) were at or below the maximum residue level (MRL), and EFSA concluded that the corresponding risk to consumer health was low [1,4]. Second, multi-residue occurrence remains common in certain fruit categories, particularly in commodities grown under conditions of high pest pressure, where several pesticide residues may be detected in a single sample. Even under these circumstances, dietary exposure assessments reported by regulatory authorities and in the peer-reviewed literature have generally remained within accepted acute and chronic health-based guidance values at the population level; however, this should not be interpreted as excluding higher exposures in susceptible subgroups, including children and high consumers, nor does it resolve persistent uncertainties regarding the cumulative assessment of chemicals sharing common modes of action [4,5,6,7,8].

Against this background, the present review addresses five decision-relevant domains in food safety. First, it summarises surveillance and compliance evidence, with emphasis on the distinction between residue detection, MRL exceedance, and toxicological relevance [5,7,8,9,10]. Second, it examines the principles governing cumulative risk assessment and mixture-oriented toxicological interpretation, including dose-addition assumptions and driver-based prioritisation [5,7,8,9,11,12,13,14]. Third, it reviews analytical strategies for multi-residue surveillance and for the identification of metabolites and transformation products, with particular emphasis on the complementary roles of targeted tandem mass spectrometry and high-resolution mass spectrometry [5,10,13,15,16,17]. Fourth, it considers regulatory divergence using the European Union, the United States, and Codex frameworks as illustrative, traceable comparators, recognising their implications for enforcement, trade, and public-facing risk communication. Fifth, it evaluates mitigation approaches for fruits not only in terms of parent-residue reduction, but also with regard to matrix realism, quality outcomes, and transformation-product reporting [5,7,8,12,18,19,20].

In contrast to earlier reviews, which have typically considered pesticide analytics, mitigation strategies, or broad health implications in isolation, the present review integrates five decision domains that are commonly addressed separately: surveillance and compliance reporting, cumulative risk assessment and mixture-oriented toxicological interpretation, analytical confidence, regulatory divergence, and mitigation evidence including transformation-product reporting. Its central contribution is a surveillance-to-decision framework for interpretation of fruit-residue data that clearly separates regulatory compliance from toxicological significance and prioritises driver-based cumulative risk assessment (CRA) over undifferentiated concern regarding residue mixtures [5,6,11,21]. The review further contributes a traceable cross-jurisdictional comparison of maximum residue levels (MRLs) and a mitigation appraisal framework that requires explicit consideration of transformation products rather than reliance on parent-compound decline alone [13,16,17,19,20,22]. Taken together, these elements are intended to move interpretation of fruit pesticide residues beyond descriptive monitoring toward a more analytically disciplined and decision-relevant synthesis.

## 2. Surveillance Evidence and Mixture-Risk Interpretation

### 2.1. Compliance Patterns and Multi-Residue Structure

Monitoring programmes in regulated markets consistently show high overall compliance with residue standards, but they also document frequent multi-residue occurrence that is directly relevant to food-safety interpretation, particularly in the context of chronic, low-dose exposure and sensitive developmental windows. Acute toxicity attributable to fruit consumption is uncommon. Important remaining uncertainties concern how repeated low-level co-exposures should be prioritised within cumulative risk assessment (CRA) and how such findings should be communicated without conflating enforcement metrics with toxicological hazard.

Official monitoring programmes in regulated markets consistently show high compliance with pesticide-residue standards, while also demonstrating that multi-residue occurrence is common in several fruit commodities. This distinction is central to food-safety interpretation: aggregate compliance metrics require toxicological context, whereas repeated low-level co-exposures may require prioritisation within cumulative risk assessment, particularly for high consumers and sensitive subpopulations. Surveillance data should therefore be used not to generate commodity-level alarms, but to identify residue patterns, recurrent co-occurrence motifs, and candidate drivers for more refined exposure assessment. Headline compliance metrics from the principal EU and US monitoring programmes are summarised in Table 1. Because annual surveillance programmes differ in reporting cycles, commodity coverage, and the level of publicly available commodity-specific detail, Table 1 is intended as a contextual summary of the official monitoring datasets used in this review rather than as a direct year-matched comparison between jurisdictions. The EU 2023 dataset was used to provide the most recent aggregate EU compliance snapshot included in the review, whereas USDA PDP 2022 was retained because the same dataset underpins the subsequent commodity-level residue and co-occurrence analyses.

In the European Union’s 2023 monitoring programme, comprising 132,793 samples, 58.0% contained no quantifiable residues, 38.3% contained residues within legal limits, and 3.7% exceeded maximum residue levels; after accounting for measurement uncertainty, 2.0% were classified as non-compliant. In the United States, the USDA Pesticide Data Program annual summary for 2022 reported that more than 99% of samples were below risk-screening benchmarks derived from EPA toxicological reference values and consumption assumptions across 10,665 samples and 23 commodities. These aggregate metrics are reassuring at the population level, but they do not describe co-occurrence within individual samples and should not be used as substitutes for targeted cumulative risk assessment or subgroup-specific exposure modelling.

#### 2.1.1. High-Frequency Residue Patterns and Implications for Cumulative Risk Assessment

To facilitate comparison across commodities and to identify likely cumulative risk drivers, the dominant residue profiles observed in selected USDA PDP 2022 fruit commodities are summarised in Table 2.

The groupings shown in Table 2 should be interpreted as surveillance-screening descriptors rather than formal cumulative assessment groups. Although the functional pesticide class is useful for identifying recurrent residue patterns and likely exposure contributors, CRA grouping should not rely on use category alone. Formal grouping requires refinement using toxicological mode of action, target-organ effects, common adverse outcomes, relative potency where available, and the quantitative contribution of each residue to total exposure.

As shown in Table 2, commodity-specific findings from the USDA PDP 2022 dataset illustrate how pest pressure and crop-protection practices generate distinct multi-residue profiles. In blueberries, 16 pesticide pairs were detected in at least 5% of samples, with a profile dominated by fungicides, notably boscalid (37.3%), azoxystrobin (34.0%), and cyprodinil (27.0%), together with the neonicotinoid insecticide acetamiprid (31.3%) [23]. Fresh grapes showed still greater complexity, with 28 pairs detected in at least 5% of samples and a prominent multi-fungicide pattern involving fluopyram (49.3%), boscalid (48.0%), cyprodinil (42.9%), tebuconazole (39.2%), and fenhexamid (37.4%) [23]. Peaches were characterised by a dominant fungicide signal, with fludioxonil detected in 87.4% of samples, accompanied by methoxyfenozide (28.2%) and spirodiclofen (26.0%) [23]. Pears reflected a mixed field and post-harvest profile, with frequent detection of pyrimethanil (63.7%), fludioxonil (50.1%), and thiabendazole (45.2%) [23]. Plums likewise showed marked dominance of fludioxonil (84.5%), with secondary contributions from methoxyfenozide (28.8%) and other fungicides [23].

These patterns are relevant not because any one residue is necessarily unsafe when considered in isolation, but because mixture exposure may be insufficiently characterised by single-chemical comparison with toxicological reference values. In this setting, monitoring data are most useful when they inform prioritisation: identifying recurrent co-occurrence motifs, dominant contributors, and plausible chemical groupings for further cumulative assessment.

#### 2.1.2. Toxicological Relevance of Co-Occurrence Patterns

The toxicological significance of co-occurrence patterns lies less in the presence of individual residues per se than in the possibility that cumulative exposure may not be adequately represented by single-substance comparisons with health-based guidance values. CRA frameworks used in both Europe and the United States generally adopt dose addition as the default assumption for chemicals with shared or convergent mechanisms of action, applying tiered methods that range from screening-level hazard indices to relative potency factor-based models [5,11,24]. EFSA has established harmonised methodologies for combined exposure assessment in which dose addition remains the default unless there is sufficient evidence to justify response addition or to support a specific interaction model, including antagonism or synergism [5]. From this perspective, monitoring-derived co-occurrence motifs are better understood not as grounds for commodity-level alarm, but as an empirical basis for driver selection, grouping hypotheses, and surveillance-linked prioritisation of mixture assessment [11].

#### 2.1.3. Sensitive Subpopulations in CRA Frameworks

Children and periods of prenatal development are commonly regarded as sensitive subpopulations in CRA because of higher food intake per kilogram of body weight, developmental toxicology considerations, and age-related differences in absorption, metabolism, and elimination [25,26]. Epidemiological evidence concerning organophosphate exposure and neurodevelopment provides a rationale for precautionary assessment assumptions. Prospective cohort studies have linked early-life exposure to organophosphate and organochlorine pesticides with adverse neurodevelopmental and behavioural outcomes, including poorer mental development and higher scores on measures of pervasive developmental disorder, inattention, and attention-deficit/hyperactivity disorder [25,27,28,29]. Prenatal exposure appears to represent a particularly important window, especially during the first and third trimesters, with reported effects on executive function, working memory, and cognitive development persisting into childhood and early adolescence [27,29,30,31]. At the same time, much of this evidence remains observational, vulnerable to residual confounding, and often dependent on short-lived or non-specific biomarkers [25,32]. Accordingly, these data are more appropriately used to justify conservative assessment assumptions than to support commodity-specific conclusions derived directly from surveillance datasets.

#### 2.1.4. Practical CRA Prioritisation Strategies

From a pragmatic perspective, monitoring-derived co-occurrence motifs can be used to focus CRA on the combinations most likely to influence cumulative exposure. To translate surveillance-derived co-occurrence patterns into operational mixture prioritisation, commodity-specific CRA-oriented motifs are summarised in Table 3. In blueberries and grapes, the dominant pattern is a fungicide cluster with secondary insecticide contributions, supporting driver-based mixture modelling centred on fungicides, with additional insecticide contributions explored through sensitivity analyses [23,24]. In peaches and plums, the presence of one dominant fungicide, fludioxonil, together with a limited number of additional residues, lends itself to an efficient driver-based strategy [23]. In pears, differentiation between post-harvest and field-applied residues is important to avoid conflating distinct exposure contexts and timings [23]. EFSA has developed specific guidance for grouping chemicals into cumulative assessment groups (CAGs) using hazard-based criteria together with exposure-driven and risk-based prioritisation approaches [5,33,34].

#### 2.1.5. Implications for Surveillance Interpretation and Risk Communication

Overall, the evidence from regulated markets supports continued fruit consumption, while also justifying disciplined surveillance, mixture-aware interpretation, and proportionate risk communication. The 30-year history of the USDA PDP shows that pesticide-residue profiles in foods change over time, particularly following the registration and uptake of new active substances, underscoring the need for continually updated monitoring data in exposure and risk assessment [23]. For food-safety decision-making, the key interpretive principle is to distinguish among compliance signals, cumulative risk uncertainty, and residue-reduction evidence, rather than extrapolating directly from occurrence data to consumer-level alarm [1,4,35]. Current EU and US approaches appear to identify most high-risk samples effectively, although methods for risk-based prioritisation continue to evolve [4,35]. In the EU, the transition from deterministic to probabilistic assessment methods has allowed more nuanced estimation of the probability of exceeding health-based guidance values across different consumer subpopulations, with overall dietary risk remaining very low for most groups [4].

## 3. Discussion

### 3.1. Analytical Detection: Enforceable at Scale Versus Discovery-Oriented Scope

**Figure 1:**
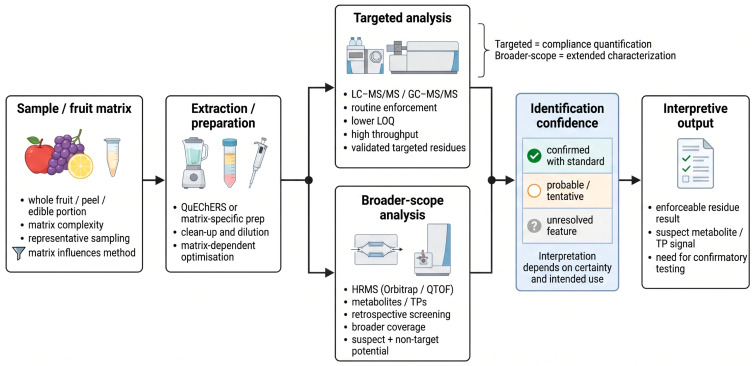
Analytical workflow for interpretation of pesticide residues in fruits. The workflow begins with matrix-specific sample preparation and proceeds through targeted LC-MS/MS or GC-MS/MS methods for routine enforcement-grade quantification, complemented where necessary by HRMS-based broader-scope screening for metabolites, transformation products, and retrospective analysis. Final interpretation depends on identification confidence, validation status, and the intended analytical use. Created in BioRender. Szarpak, Ł. (2026) https://BioRender.com/xkllpbs.

Maximum residue levels (MRLs) are intended to represent the highest residue concentration expected when a pesticide is used in accordance with good agricultural practice (GAP), and they are accepted only where dietary exposure estimates remain below health-based guidance values such as the acceptable daily intake (ADI) and, where relevant, the acute reference dose (ARfD) [36,37]. MRLs should therefore be understood primarily as enforcement benchmarks rather than direct indicators of safety [20,22,36]. Residues below the MRL are generally considered unlikely to present a health concern under the assumptions embedded in regulatory assessment, whereas residues above the MRL do not in themselves establish toxicological harm [36,37,38]. More commonly, they indicate a possible departure from GAP and require case-specific dietary risk assessment that takes into account consumption patterns and potentially vulnerable subgroups [36,38]. This separation between hazard characterisation (ADI/ARfD) and exposure control through enforcement limits (MRLs) makes pesticide-residue risk interpretation less intuitively transparent than for many other contaminants in food [36]. Residue concentrations at or below the MRL are not expected to result in intakes exceeding the ADI or ARfD, whereas concentrations above the MRL require individual assessment to determine whether health-based guidance values could in fact be exceeded [36,37]. This distinction is fundamental to the sound interpretation of monitoring data and to proportionate risk communication. The analytical logic linking enforcement-grade quantification, broader-scope screening, and identification-confidence categories is summarised in Figure 1.

Within this framework, analytical strategy matters because enforcement-grade surveillance and discovery-oriented profiling serve related but distinct purposes. High-resolution mass spectrometry (HRMS), most commonly based on Orbitrap or quadrupole time-of-flight (QTOF) platforms, broadens analytical scope by supporting targeted, suspect-screening, and non-targeted workflows [7,39]. This is particularly valuable for the detection of metabolites and transformation products and for retrospective interrogation of archived datasets [7,39]. Owing to its mass-resolving power, HRMS requires different analytical concepts, validation approaches, and interpretive criteria for screening, identification, and quantification in complex food matrices than those used for triple-quadrupole platforms [7]. In routine residue control, however, triple-quadrupole MS/MS generally remains the more sensitive tool for quantification, and HRMS—if insufficiently validated—may be associated with higher quantification limits and a greater likelihood of false negatives at trace concentrations [40,41,42].

Comparative studies consistently indicate that, for quantitative purposes, triple-quadrupole instruments typically achieve lower limits of quantitation (LOQs) than HRMS operated in full-scan mode [40,42]. In one comparison of GC-Q-Orbitrap and GC-triple-quadrupole methods, 86% of molecules showed lower limits of detection with GC-Q-Orbitrap; however, in exposome-type applications, the median LOQ for HRMS remained approximately ten-fold higher than that observed with triple-quadrupole instrumentation [40,41]. Recent developments in HRMS acquisition strategies, particularly targeted MS^2^ (tMS^2^) combined with full-scan acquisition and all-ion fragmentation, have improved both sensitivity and identification performance, making HRMS increasingly competitive for routine quantitative applications [39]. Even so, the analytical roles of the two platforms are not interchangeable. Triple-quadrupole MS/MS remains the preferred platform where high-throughput, low-LOQ, enforcement-grade quantification is the primary objective, whereas HRMS is especially advantageous where broader chemical scope, metabolite discovery, or retrospective interpretability is required.

Given these complementary strengths and limitations, HRMS-based reporting should communicate identification confidence explicitly and conservatively. At a minimum, reports should distinguish among: (i) confirmed identifications supported by an authentic reference standard together with concordant retention time and MS/MS matching; (ii) probable or tentative identifications supported by accurate mass and spectral-library evidence but lacking confirmation with a reference standard; and (iii) unresolved features requiring further structural characterisation [7,39]. Such transparency reduces the risk of false-positive interpretations and supports analytically and regulatorily defensible conclusions. Use of structured confidence frameworks—for example, levels 1, 2a, 2b, 3, 4, and 5—also promotes greater consistency across laboratories and studies [39].

### 3.2. Regulatory Divergence: EU Versus US Versus Codex

Differences in pesticide MRLs and tolerances across the European Union, the United States, and the Codex Alimentarius framework have practical implications for both international trade and public-facing risk communication. These limits are derived from authorised agricultural use patterns and dietary exposure modelling anchored to toxicological reference values, but they are not themselves toxicity thresholds [20,22,36]. The Codex Alimentarius Commission was established to develop harmonised international food standards intended both to protect consumer health and to promote fair practices in food trade, with Codex MRLs based on recommendations issued by the FAO/WHO Joint Meeting on Pesticide Residues (JMPR) [20,22]. Illustrative examples of EU, US, and Codex divergence for selected citrus residues are summarised in Table 4. The EU legal source trail supporting the MRL values used in Table 4 is provided in Supplementary Table S1. Corresponding US CFR and Codex traceability records are provided in Supplementary Table S2. These examples should be interpreted as auditable regulatory case examples rather than as a comprehensive global mapping of pesticide-residue legislation or MRL systems.

In practice, however, cross-jurisdictional divergence remains substantial and predictably generates both trade friction and inconsistent messaging. The EU may apply lower MRLs and, in some settings, default limits of 0.01 mg/kg when essential supporting data are lacking, whereas US tolerances may be higher for the same active substance in the same commodity [12,18,19]. Codex MRLs are intended to facilitate international harmonisation, but they are not adopted uniformly across jurisdictions [18,19,20,22]. In broad terms, US tolerances tend to exceed EU MRLs, and among pesticide classes, fungicides generally show the highest residue limits, followed by insecticides and herbicides [18]. As a result, shipments compliant under US or Codex standards may nevertheless fail under EU rules, or conversely, despite no meaningful difference in consumer risk when exposure is evaluated against health-based guidance values [12,19].

This divergence is not trivial. A global analysis covering 114 nations reported that nearly 30% of computed pesticide theoretical maximum daily intake (TMDI) values exceeded corresponding ADI values, while also showing that many countries lack common pesticide MRLs for widely consumed foods [19]. The distribution of TMDI values was markedly right-skewed, with clustering at the low end driven in part by the stricter MRL structure applied in the EU [19]. In addition, physicochemical characteristics—including aromatic proportion, non-carbon proportion, and water solubility—and crop type have been shown to explain up to 50% of the variation in residue limits across jurisdictions [18]. These findings underscore that MRL divergence reflects not only toxicological interpretation but also regulatory culture, data requirements, agronomic practice, and policy choice.

For consumers, regulators, and trade stakeholders, this regulatory heterogeneity creates a communication problem because the same residue finding may be classified differently across jurisdictions. In this section, the relevant issue is therefore not the toxicological meaning of a single MRL exceedance, which has been addressed above, but the practical consequence of divergent legal limits for enforcement, trade, and public confidence. Divergence in MRLs may function as a non-tariff barrier to trade and may erode stakeholder confidence when legal limits are interpreted without sufficient regulatory context [12,36,43].

Divergence in MRLs functions as a non-tariff barrier to trade and may erode stakeholder confidence when enforcement limits are misconstrued as health-risk thresholds. This reinforces the need for precise, disciplined communication that separates compliance criteria from toxicological risk [12,22].

### 3.3. Human Biomonitoring as Contextual Evidence for Fruit-Residue Assessment

Human biomonitoring is not a primary tool for surveillance of pesticide residues in fruit, but rather a contextual source of evidence that can inform exposure interpretation at the population level. Its principal strengths lie in capturing aggregate internal dose, documenting broad shifts after dietary or regulatory change, and supporting epidemiological investigation [44,45,46]. Its limitations are equally important: biomarkers are often non-specific, short-lived, and poorly suited to commodity-level source attribution or enforcement decision-making [47,48].

Across pesticide classes, commonly used biomarkers include dialkylphosphates (DAPs) and selected compound-specific metabolites such as 3,5,6-trichloro-2-pyridinol (TCPy) and para-nitrophenol for organophosphates; 3-phenoxybenzoic acid (3-PBA) together with cis- and trans-3-(2,2-dichlorovinyl)-2,2-dimethylcyclopropane carboxylic acid (DCCA) for pyrethroids; ethylenethiourea (ETU) for dithiocarbamates; and selected parent compounds or metabolites—including 6-chloronicotinic acid, imidacloprid, acetamiprid, clothianidin, and desnitro-imidacloprid—for neonicotinoids [49,50,51,52]. By contrast, consensus biomarkers remain limited for many fungicides. This uneven degree of biomarker maturity across pesticide classes constrains direct comparison and reinforces the point that biomonitoring evidence is stronger for some chemical groups than for others [49,51,53]. Recent analytical methods based on solid-phase extraction and liquid chromatography–tandem mass spectrometry (LC-MS/MS) permit simultaneous determination of multiple pesticide classes and metabolites in urine, with limits of quantitation ranging from 0.1 to 16 pg/mL and detection frequencies exceeding 50% for several organophosphate and pyrethroid metabolites in general-population samples [51,53,54].

Intervention and cohort studies show that urinary biomarker concentrations can respond to short-term dietary change and can be linked to broader epidemiological endpoints; however, these signals generally reflect aggregate exposure across multiple pathways rather than fruit-specific intake [44,47,48]. Validation studies using pesticide residue burden scores (PRBS), derived from food-frequency questionnaires together with USDA Pesticide Data Program surveillance data, have demonstrated significant associations between estimated dietary pesticide intake from fruits and vegetables and urinary biomarker concentrations [44,45,46,48]. The molar sum of urinary pesticide biomarkers has been reported to be 21% higher for each additional daily serving of high-pesticide fruits and vegetables and 10% lower per additional daily serving of low-pesticide fruits and vegetables [47]. Even so, duplicate-diet studies highlight important interpretive complications: some urinary metabolites are already present in the consumed diet as preformed metabolites, which may lead to overestimation of exposure to the parent pesticide when biomarker data are interpreted simplistically [47]. Quantitative comparisons also indicate that non-dietary background exposure, including household use and environmental sources, limits the specificity of biomarkers for dietary source attribution [47,53].

For these reasons, biomonitoring functions primarily as contextual support for exposure plausibility, temporal trends, and subgroup sensitivity rather than as a substitute for occurrence data, commodity-level surveillance, or analytical enforcement workflows [44,45,46]. Within the logic of this review, human biomonitoring therefore remains a secondary evidentiary stream: useful for triangulating exposure narratives and identifying settings in which greater toxicological caution may be justified, but not an appropriate organising framework for assessment of pesticide residues in fruit.

### 3.4. Decontamination and Residue Reduction: Study Type and Transformation-Product Reporting

#### 3.4.1. Sodium Bicarbonate Washing

Sodium bicarbonate washing, typically applied as approximately 1% solutions for 10–15 min, can reduce selected pesticide residues more effectively than water alone, particularly when residues are predominantly superficial [15,16]. To provide a comparative overview before method-specific discussion, the mitigation evidence base is summarised in Supplementary Table S3, including typical conditions, parent-residue reduction, quality endpoints, and the extent of transformation-product reporting. In controlled experiments on apples, baking soda solutions achieved substantially greater removal of certain pesticides than either tap water or commercial bleach-based washing solutions [15]. Using a 10 mg/mL NaHCO_3_ solution, complete removal of surface residues required 12 min for thiabendazole and 15 min for phosmet after 24 h pesticide exposure [15]. Importantly, however, removal was incomplete once residue penetration into the fruit matrix had occurred. After the same 24 h exposure, 20% of the applied thiabendazole and 4.4% of the applied phosmet had penetrated into the apple, with the systemic compound thiabendazole reaching approximately fourfold greater depth within the peel than the non-systemic compound phosmet [15]. In this setting, NaHCO_3_ appears to contribute not only through mechanical washing but also through partial chemical degradation of the parent compounds, thereby facilitating residue removal [15].

Evidence from comparative studies across multiple commodities supports the broader conclusion that alkaline washing solutions can reduce pesticide residues effectively, particularly when contact times exceed 15 min [16,55,56]. In bell peppers, sodium bicarbonate achieved removal efficiencies of 60% to 81% for fungicide residues, although this was accompanied by deterioration in colour quality [55]. In sweet cherries, a 5% sodium bicarbonate solution reduced pesticide residues effectively, but at the cost of greater mass loss and reduced firmness relative to distilled water or vinegar treatments [56]. As expected, efficacy is compound-dependent. Pyrethroids generally appear more removable, whereas chlorpyrifos has repeatedly proved relatively resistant to washing-based decontamination [16,57]. Taken together, these findings indicate that sodium bicarbonate washing should be interpreted as a surface-decontamination strategy with compound- and matrix-dependent performance rather than as a universal residue-removal approach.

#### 3.4.2. Peeling

Where feasible, peeling is consistently the most effective single intervention for residue reduction, because it physically removes both surface residues and part of the penetrated fraction within the peel [13,15,17]. For this reason, peeling often achieves larger reductions than washing alone, especially for compounds that have migrated beneath the immediate fruit surface. The practical limitation is equally clear: removal of the peel also removes peel-associated fibre and phytochemicals, thereby reducing nutritional value [15]. In apples, for example, sodium bicarbonate washing reduces residues mainly from the surface, whereas peeling is more effective for residues that have penetrated into the peel tissue; the benefit in residue reduction is therefore counterbalanced by loss of bioactive compounds concentrated in the skin [15]. From a food-quality perspective, peeling is thus highly effective but not without cost, and its relevance depends on the commodity, the expected residue distribution, and the importance of peel-associated nutritional attributes.

#### 3.4.3. Ozone Treatment

Ozone-based treatments, including ozonated water and ozone microbubble systems, can produce moderate to high reductions in measured parent pesticide residues, although efficacy depends strongly on pesticide identity, fruit matrix, and treatment conditions. In bell peppers, water continuously bubbled with ozone at 3 mg/L achieved fungicide-residue reductions ranging from 67% to 87% [55]. At a lower concentration of 1 mg/L, fungicide removal remained substantial, ranging from 53% to 75%, while fruit quality was better preserved during 13 days of storage [55]. Ozone microbubble treatment has also shown measurable effects in other fruits, with residue reductions of 51–65% in strawberries, 51–59% in cherries, and 24–70% in apricots after prolonged washing times of 18 min; vitamin C content in these studies remained largely unchanged [58].

The interpretive issue, however, is not simply whether parent residues decline, but what is formed during treatment. Because ozone is highly reactive, transformation-product (TP) formation is plausible and, in many cases, expected. Available studies and reviews increasingly recognise this point, emphasising the need for improved analytical characterisation of degradation products rather than assuming that a decrease in parent compound concentration necessarily equates to a reduction in toxicological risk [59]. Ozone treatment has shown considerable utility in removing pesticide residues from soil, water, and food systems, whether used alone or in combination with other methods [59]. Nevertheless, major uncertainties remain, particularly regarding the identification, persistence, and toxicological relevance of ozone-derived by-products [59]. For this reason, ozone should be interpreted as a promising decontamination tool whose apparent efficacy depends not only on parent-residue removal but also on the adequacy of TP characterisation.

#### 3.4.4. Cold Plasma

Cold plasma is an emerging non-thermal technology that has shown rapid and, in some pilot studies, substantial reductions in parent pesticide residues [13,14,17]. Its principal attraction lies in the possibility of efficient decontamination without the thermal burden associated with conventional processing. At the same time, the central unresolved issue is the identity and toxicological significance of the resulting intermediates and transformation products, which remain incompletely characterised [14,17]. Current reviews consistently note that residue-reduction data alone are insufficient and that harmonised TP reporting, together with toxicological evaluation, is required if the technology is to be interpreted confidently from a food-safety perspective [14,17].

Mechanistically, cold plasma treatment involves oxidative processes driven by reactive species, including hydroxyl radicals, together with photo-oxidative pathways that partially overlap with those described for ozone and ultraviolet-based treatments [14]. In some experimental settings, rapid or even apparently complete degradation of parent residues has been reported, supporting continued interest in scale-up and potential commercial application [14]. However, the toxicological properties of intermediate and degradation products generated during plasma processing have received comparatively limited attention [14]. For practical use, this gap is not peripheral but central. The safety of end products formed during plasma-mediated degradation must be addressed explicitly, alongside identification of major intermediates, clarification of the most relevant reactive plasma species, assessment of matrix effects, and evaluation of reactor energy efficiency and process economics [14]. Until such issues are addressed systematically, cold plasma should be regarded as analytically promising but toxicologically under-characterised.

#### 3.4.5. Electrolysed Water

Electrolysed water, whether acidic or alkaline and typically applied for 5–25 min, has demonstrated substantial reductions in several pesticide classes in fresh and fresh-cut produce, often with limited adverse effects on texture under appropriately optimised conditions [13,16,60]. As with other decontamination methods, however, efficacy is matrix- and protocol-dependent. In kumquat and cucumber, alkaline electrolysed water at high pH showed greater residue-removal capacity than other washing solutions [16]. Recent reviews further indicate that electrolysed water technologies can inhibit microbial growth, reduce browning, extend shelf life, and provide a non-thermal means of lowering pesticide residues [60]. These broader technological advantages make electrolysed water attractive not only as a residue-reduction strategy but also as part of integrated post-harvest quality control.

There is also increasing interest in combining electrolysed water with complementary technologies, including ozone, ultraviolet light, ultrasound, and high-voltage electrostatic fields, to enhance decontamination performance and broader produce quality outcomes [60]. Even so, the same interpretive caution applies: reductions in measured parent residues do not, on their own, establish toxicological benefit unless degradation pathways and resulting products are also characterised adequately.

#### 3.4.6. Interpretive Limitations and Reporting Needs

Across decontamination methods, the principal interpretive limitations are strikingly consistent. First, efficacy varies substantially according to pesticide class, fruit type, residue localisation, and treatment protocol. Second, study designs are highly heterogeneous, which limits direct comparability and weakens external validity. Third, and most importantly, transformation products are only rarely characterised in a systematic way and even less often evaluated toxicologically [13,14,17,61]. As a result, the apparent success of a decontamination method is still too often judged by a decline in parent-residue concentration alone.

Recent reviews increasingly call for harmonised study designs that integrate three elements: quantitative parent-residue reduction, transparent TP reporting, and relevant quality outcomes such as texture, colour, nutrient retention, and storage stability [13,17,61]. This integrated reporting framework is particularly important for emerging technologies such as cold plasma, ultrasound, electrolysed water, and pulsed electric field treatment, which may in some settings reduce pesticide content more effectively than conventional methods without obvious visible damage to produce [13]. However, the extent of residue dissipation and the safety of the resulting treated food remain highly dependent on equipment design, process parameters, produce surface characteristics, treatment conditions, and pesticide chemistry [13]. For food-safety interpretation, the key point is therefore not simply whether a method lowers the measured parent residue, but whether it does so under realistic conditions, with acceptable quality trade-offs, and with sufficient analytical attention to the products formed in the process.

### 3.5. Toward an Integrated Surveillance-to-Decision Framework

#### 3.5.1. The Central Interpretive Challenge

The principal interpretive challenge is not the detection of pesticide residues itself, but the persistent tendency to discuss monitoring, analytics, regulation, cumulative risk assessment (CRA), and mitigation as if they were separate domains. For fruit residues, these domains are not independent; they form a sequential decision pathway. Surveillance defines occurrence and co-occurrence patterns; analytical workflows determine what can be measured with sufficient confidence; CRA identifies the residues and combinations that matter most toxicologically rather than treating all detections as equally informative; regulatory comparison distinguishes legal divergence from toxicological significance; and mitigation evidence becomes decision-relevant only when apparent parent-residue reduction is interpreted in light of matrix realism and uncertainty regarding transformation products (TPs).

**Figure 2:**
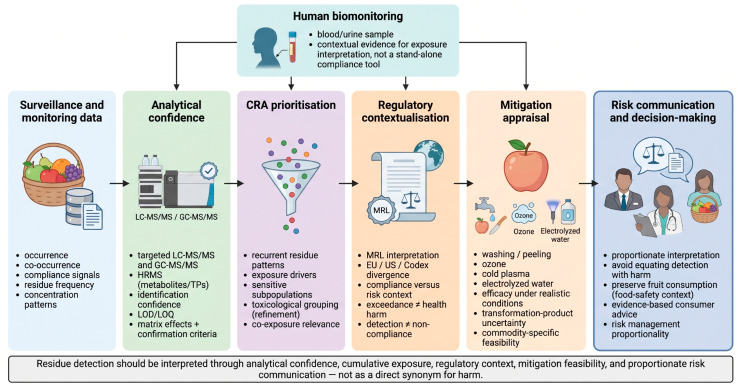
Integrated surveillance-to-decision framework for interpretation of pesticide residues in fruits. Surveillance data define occurrence, co-occurrence, and compliance signals. Analytical workflows determine what can be measured with sufficient confidence, including targeted residues, metabolites, and transformation products. These data then support cumulative risk assessment prioritisation, with emphasis on recurrent exposure drivers and sensitive subpopulations. Regulatory contextualisation distinguishes legal compliance from toxicological interpretation and accounts for divergence across EU, US, and Codex frameworks. Mitigation appraisal considers residue reduction, matrix realism, quality outcomes, and transformation-product uncertainty. The final step is proportionate risk communication and food-safety decision-making. Created in BioRender. Szarpak, Ł. (2026) https://BioRender.com/t9ehsbj.

The proposed surveillance-to-decision framework is summarised in Figure 2. It illustrates how residue findings should move from surveillance and analytical confirmation through cumulative risk prioritisation, regulatory contextualisation, mitigation appraisal, and finally proportionate risk communication, with human biomonitoring positioned as contextual evidence rather than as a stand-alone compliance tool.

#### 3.5.2. MRL Exceedances: Compliance Versus Toxicity

Within the proposed surveillance-to-decision framework, MRL exceedances are treated as entry points for interpretation rather than endpoints. Their relevance depends on the magnitude and persistence of the finding, consumption context, co-occurring residues, and applicable toxicological reference values. The purpose of this step is to prevent two opposing errors: overstating hazard from an isolated compliance signal or overlooking exposure patterns that may become relevant because of co-occurrence, high consumption, or subgroup vulnerability.

#### 3.5.3. Driver-Based Cumulative Risk Assessment

**Figure 3:**
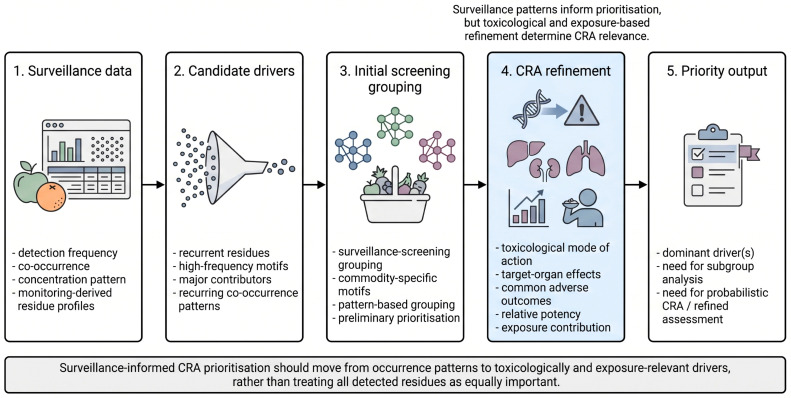
Stepwise workflow for surveillance-informed cumulative risk assessment (CRA) prioritisation. Monitoring data first identify recurrent residues, co-occurrence motifs, and candidate exposure drivers. These signals support initial screening-level grouping but require further toxicological refinement using mode of action, target-organ effects, common adverse outcomes, relative potency, and exposure contribution before formal CRA grouping or prioritisation decisions are made. Created in BioRender. Szarpak, Ł. (2026) https://BioRender.com/5fs09sj.

The operational logic of surveillance-informed, driver-based CRA prioritisation is summarised in Figure 3. Because multi-residue findings are common, the most informative step after surveillance is driver-based CRA grounded in commodity-specific co-occurrence motifs and analytically reliable residue data [5,6,21]. This approach avoids both under-interpretation of mixture exposure and over-interpretation of trace detections with limited toxicological relevance. CRA is inherently resource-intensive; accordingly, both toxicological evaluation and intake estimation are best approached in a tiered manner [19]. Inclusion of compounds within a cumulative assessment group (CAG) should be based on explicit criteria that allow progressive refinement, including chemical structure, pesticidal mechanism of action, target organ, and toxic mode of action [5].

Probabilistic cumulative dietary risk assessments performed in several European countries have generally indicated that cumulative chronic dietary exposure is unlikely to constitute a health risk for most consumer groups, with hazard indices or total margins of exposure typically remaining below regulatory thresholds of concern [6,21]. Nevertheless, important uncertainty persists for some CAGs, particularly those relevant to the nervous system and thyroid function, and under current methodological conditions, a potential health risk cannot be excluded completely [6]. Among the most influential drivers identified in these assessments are chlorpyrifos and the dithiocarbamate group [6].

#### 3.5.4. Mitigation Appraisal

The same interpretive discipline should be applied to mitigation evidence. Washing, peeling, ozone, cold plasma, and related interventions should not be judged solely by the decline in parent-compound concentration, but by whether they achieve decision-relevant reductions under realistic conditions, preserve food quality where relevant, and address plausible TP formation [14,61]. Recent work also suggests that combined methods, selected according to the mechanism of decontamination and suitability for a given fruit or vegetable, may offer a more effective way to reduce both microbial contamination and pesticide residues while limiting treatment costs and improving overall food-safety performance [61].

#### 3.5.5. Risk–Benefit Communication

Taken together, the evidence supports a surveillance-to-decision model in which residue interpretation proceeds from detection to prioritisation, then to regulatory and toxicological contextualisation, and finally to mitigation appraisal. The value of this model lies in its ability to reduce common category errors: equating exceedance with toxicity, presence with risk, or parent-residue removal with hazard elimination. It therefore offers a more coherent basis for food-safety interpretation of pesticide residues in fruit.

Risk communication should remain anchored in the broader dietary context. Evidence-based communication consistently indicates that the health benefits of fruit and vegetable consumption substantially outweigh pesticide-related risks [62,63,64,65,66,67,68]. In one analysis, for each estimated cancer case attributed to pesticide exposure, at least 88 cases were considered prevented through fruit and vegetable consumption on the basis of the population etiological fraction for diet-related cancer prevention [62]. Chronic health risks associated with pesticide exposure through produce have generally been estimated to be low, whereas the health benefits of fruit and vegetable intake are much greater, although uncertainty remains and risk estimates are not zero [62,69,70,71,72]. The most reliable strategies for materially reducing dietary pesticide risk include shifting relatively high-risk fruits and vegetables toward organic production and, more broadly, reducing dependence on pesticides overall, particularly those considered high risk [63,73,74,75].

### 3.6. Limitations and Research Priorities

This review is narrative rather than systematic. Although it prioritises primary regulatory sources and higher-quality reviews, it was not designed to be exhaustive and is therefore susceptible to selection bias. In addition, residue monitoring programmes are built primarily to support compliance assessment and exposure modelling rather than to measure biologically effective dose. Translation of residue findings into individual-level risk is therefore necessarily limited. A further limitation is the restricted geographic scope of the regulatory comparison. The review focuses on EU, US, and Codex frameworks because these were used for auditable source tracing in the citrus MRL examples. Major jurisdictions such as China, Japan, Australia, and New Zealand were not analysed in detail; inclusion of these systems would require a broader comparative regulatory review beyond the scope of the present decision-oriented synthesis.

Several research priorities emerge repeatedly from the literature. First, mixture-aware exposure assessment, including driver-based CRA, should be integrated more routinely into the interpretation of surveillance data [5,6,11,76]. Second, transformation products generated during oxidative or other decontamination processes require more standardised reporting and more explicit hazard contextualisation [14]. Third, risk-communication frameworks should be developed that preserve fruit intake while addressing recurrent misinterpretation of residue findings [62,63,77,78,79].

Additional priorities include the development of harmonised data sources to support robust refinement in cumulative risk assessment; the establishment of criteria for prioritising CAGs on the basis of monitoring frequency, extent of use, exposure relative to toxicological reference values, and the number of compounds within a group; and more rigorous investigation of how emerging decontamination technologies affect nutritional and organoleptic quality under conditions that achieve meaningful pesticide reduction. There is also a clear need to characterise degradation pathways for different pesticides under different treatment technologies, both to improve applicability and to clarify whether apparent residue reduction corresponds to toxicological benefit [80,81,82].

## 4. Methods

### 4.1. Review Design and Scope

This article was developed as a critical integrative review of pesticide residues in fruits, with a deliberate focus on five domains that are central to food-safety interpretation: (i) surveillance and compliance reporting, (ii) cumulative risk assessment (CRA) and mixture-oriented toxicological interpretation, (iii) analytical strategies for targeted and broader-scope residue detection, (iv) regulatory divergence in maximum residue levels (MRLs) across jurisdictions, and (v) mitigation approaches, including the extent to which transformation products (TPs) are considered. The review was designed on the premise that these domains are often discussed separately, although in practice they jointly determine how fruit-residue findings should be interpreted.

Because the purpose of the article was interpretive synthesis across regulatory, analytical, toxicological, and mitigation evidence, rather than quantitative pooling of results for a single predefined outcome, the review was conducted as a structured narrative review rather than a systematic review or meta-analysis.

### 4.2. Regulatory and Surveillance Sources

Primary regulatory and surveillance documents were treated as the core evidence base for interpretation of compliance data and residue-limit frameworks. These included annual pesticide-residue monitoring reports published by the European Food Safety Authority (EFSA), annual summaries from the United States Department of Agriculture (USDA) Pesticide Data Program (PDP), EU analytical quality-control and validation guidance (SANTE), Regulation (EC) No 396/2005 and relevant implementing regulations, United States Environmental Protection Agency (EPA) tolerances listed in 40 CFR Part 180, and the Codex Alimentarius pesticide-residue database.

These materials were used preferentially whenever the review addressed legal status, compliance interpretation, analytical validation expectations, or cross-jurisdictional MRL comparison. For the citrus examples presented in Table 4, source tracing was performed directly against the relevant legal and regulatory records. EU source trails were compiled in Supplement Table S1, with corresponding US and Codex traceability collated in Supplement Table S2. The regulatory comparison was deliberately restricted to the European Union, the United States, and Codex Alimentarius because these frameworks provided directly traceable legal and database records for the illustrative MRL comparisons used in this review. The analysis was not intended to provide a comprehensive global comparison of pesticide-residue legislation. Other major jurisdictions, including China, Japan, Australia, and New Zealand, were therefore outside the formal regulatory comparison and are acknowledged as important areas for future comparative work.

### 4.3. Literature Search Strategy

To identify peer-reviewed literature relevant to the interpretive aims of the review, structured searches were conducted in PubMed and Scopus from database inception to 30 January 2026. These databases were selected because together they provide broad coverage of food analysis, analytical toxicology, exposure science, regulatory science, biomonitoring, and nutrition-related public-health literature. In addition, backward and forward citation tracking was used selectively where important reviews, methodological papers, or regulatory documents indicated further relevant sources.

The search strategy combined terms related to fruit commodities, pesticide residues, mixture exposure, analytical methods, mitigation technologies, and biomonitoring. A representative search structure was: (“fruit” OR “apple” OR “berries” OR “citrus” OR “grapes” OR “peach” OR “pear” OR “plum”) AND (“pesticide*” OR “residue*”) AND (“multi-residue” OR “mixture” OR “cumulative” OR “co-occurrence”) AND (“LC-MS/MS” OR “GC-MS/MS” OR “QuEChERS” OR “HRMS” OR “high-resolution mass spectrometry” OR “Orbitrap” OR “QTOF”).

This core strategy was supplemented, where relevant, with additional terms for mitigation methods, including washing, peeling, ozone, cold plasma, electrolysed water, ultrasound, and pulsed electric field, and for biomonitoring, including urinary metabolites and commonly used pesticide biomarkers.

Searches were refined iteratively during manuscript development when clarification was needed for specific analytical, toxicological, or mitigation issues. In these instances, emphasis was placed on methodological relevance and source authority rather than on maximising the number of retrieved citations.

### 4.4. Eligibility Criteria

Records were considered eligible if they made a direct contribution to one or more of the predefined review domains. Eligible sources included: (i) regulatory reports, legal texts, and official technical guidance relevant to surveillance, validation, or MRL interpretation; (ii) systematic reviews, narrative reviews of clear methodological value, consensus statements, and guidance documents relevant to CRA, HRMS, biomonitoring, or mitigation; and (iii) primary studies in fruit or other clearly defined food matrices reporting interpretable data on residue occurrence, co-occurrence, analytical performance, mitigation, degradation, or biomonitoring associations relevant to dietary pesticide exposure.

For analytical-method studies, preference was given to papers that provided sufficient methodological detail to permit judgement of analytical reliability, including matrix, extraction procedure, instrument platform, and at least basic validation characteristics such as detection or quantification limits. For mitigation studies, emphasis was placed on realistic fruit matrices, clearly specified treatment conditions, and reporting of parent-residue reduction together with any information on quality endpoints or TP formation. For biomonitoring studies, inclusion was based on relevance to exposure interpretation, subgroup sensitivity, or diet-related biomarker patterns, even when direct commodity-specific attribution was not possible.

Editorials without substantive data, studies lacking clear relevance to fruit-residue interpretation, and reports with insufficient methodological detail to support analytical or toxicological interpretation were excluded.

### 4.5. Study Selection and Source Prioritisation

Titles and abstracts were screened for relevance to the five domains of interest. Full texts were then examined with particular attention to methodological clarity, interpretive value, and relevance to the surveillance-to-decision framework used in this review. Source selection was guided not simply by recency, but by relevance and authority. Recent evidence was prioritised where the subject concerned evolving regulatory reporting, newer analytical platforms, or emerging mitigation technologies; however, older sources were retained when they remained methodologically foundational or continued to define current regulatory or technical practice.

When several sources addressed the same issue, priority was generally given to official regulatory or legal documents, followed by methodological guidance and consensus documents, then by higher-quality reviews, and finally by primary studies. This was performed to ensure that statements regarding compliance, legal residue limits, and analytical expectations remained anchored to the most authoritative evidence available.

### 4.6. Data Extraction

Data extraction was performed in a structured manner according to source type. From surveillance reports, the main variables extracted were sample numbers, frequencies of non-detects and quantifiable residues, exceedance and non-compliance rates, and commodity-level detection patterns relevant to co-occurrence analysis. From legal and database sources, jurisdiction-specific MRLs or tolerances were extracted together with commodity definitions and source trails. From analytical studies, we extracted matrix type, sample-preparation approach, instrument platform, principal validation characteristics, and whether metabolites or TPs were addressed. From CRA and toxicology sources, we extracted grouping logic, assumptions regarding dose addition, treatment of cumulative assessment groups, and identified drivers of cumulative exposure or uncertainty. From biomonitoring studies, extraction focused on biomarker type, biological matrix, exposure context, and the extent to which the findings informed interpretation of dietary rather than non-dietary exposure. From mitigation studies, the extracted variables included treatment type, operating conditions, fruit matrix, reported parent-residue reduction, quality effects, and the presence or absence of TP assessment.

### 4.7. Synthesis Approach

The evidence was synthesised narratively and comparatively rather than quantitatively pooled. The objective was not to summarise one outcome measure across studies, but to integrate heterogeneous evidence streams into a coherent interpretive framework. For that reason, synthesis was organised around five practical questions: what surveillance programmes show about occurrence and co-occurrence; what can be measured with confidence using current analytical workflows; which residue patterns are most relevant for driver-based CRA; how cross-jurisdictional MRL differences should be interpreted; and under what conditions apparent residue reduction becomes meaningful from a food-safety perspective.

To improve transparency of this synthesis, tabulated summaries were constructed for headline compliance metrics, dominant residue profiles, CRA-oriented co-occurrence motifs, traceable EU/US/Codex MRL comparisons, and mitigation evidence stratified by typical conditions and TP reporting.

### 4.8. Limitations and Research Priorities

This review has several limitations that should be considered when interpreting its conclusions. First, it was designed as a critical integrative review rather than as a formal systematic review or meta-analysis. It was therefore not intended to provide an exhaustive inventory of all publications on pesticide residues in fruits. No single risk-of-bias instrument was applied across all included records because the evidence base was heterogeneous and included legal texts, surveillance reports, analytical-method papers, cohort studies, regulatory guidance, and experimental mitigation studies. Methodological robustness was addressed through explicit source prioritisation, direct use of primary regulatory materials for compliance and maximum residue level (MRL) interpretation, and selective emphasis on analytically and interpretively informative sources.

Second, because this was not a PRISMA-based systematic review, searches were not managed to generate a formal record-flow count of all records retrieved, screened, excluded, and included. The final manuscript cites 97 sources selected from regulatory and surveillance documents, legal and technical guidance, methodological papers, higher-quality reviews, and primary studies directly relevant to the five predefined review domains. This limits reproducibility in comparison with a systematic review, and the manuscript should therefore be read as a structured, decision-oriented synthesis rather than as a comprehensive systematic evidence map.

Third, the surveillance datasets discussed in this review are subject to inherent design and reporting biases. Official monitoring programmes are primarily designed for compliance assessment, enforcement prioritisation, and exposure modelling, not for unbiased estimation of all consumer exposures. Sampling may be influenced by commodity selection, import patterns, national control priorities, previous exceedance history, analytical target lists, reporting thresholds, and laboratory capacity. Consequently, monitoring data can identify relevant occurrence and co-occurrence patterns, but they should not be interpreted as fully representative population-level exposure datasets.

Fourth, the monitoring evidence is time sensitive. Residue profiles may change as pesticide authorisations, pest pressure, agricultural practice, post-harvest treatment, analytical scope, and limits of detection or quantification evolve. The EU and USDA datasets used in this review therefore provide decision-relevant surveillance examples for the reporting periods analysed, but they should not be treated as fixed or permanent residue patterns. Future assessments should continue to update co-occurrence motifs and cumulative risk priorities as newer monitoring data become available.

Fifth, the regulatory comparison has a restricted geographic scope. The review focuses on the European Union, the United States, and Codex Alimentarius because these frameworks provided directly traceable legal and database records for the illustrative MRL comparisons used in the manuscript. Other major jurisdictions, including China, Japan, Australia, and New Zealand, were not analysed in detail. Their inclusion would require a broader comparative regulatory review and may further refine the interpretation of global MRL divergence, trade implications, and harmonisation challenges.

Sixth, cumulative risk assessment remains constrained by uncertainty in grouping logic and exposure modelling. Surveillance-screening descriptors, such as recurrent fungicide-dominated residue patterns, are useful for identifying candidate exposure drivers, but they should not be interpreted as formal cumulative assessment groups. Formal grouping requires refinement according to toxicological mode of action, target-organ effects, common adverse outcomes, relative potency where available, and quantitative exposure contribution. Subgroup-specific exposure assessment also remains limited by uncertainty in consumption patterns, co-exposure scenarios, and assumptions for sensitive populations.

Seventh, the mitigation evidence base remains uneven. Many studies of washing, peeling, ozone, cold plasma, electrolysed water, and related technologies report reductions in parent pesticide concentrations, but transformation products are often incompletely identified and rarely evaluated toxicologically. This is a major limitation because apparent parent-residue reduction does not necessarily equate to hazard reduction when degradation products are unknown, persistent, or insufficiently characterised. In addition, mitigation studies vary substantially in fruit matrix, treatment conditions, contact time, residue localisation, quality endpoints, and analytical follow-up, which limits direct comparison and external validity.

Finally, human biomonitoring provides useful contextual evidence for aggregate exposure, temporal trends, and subgroup sensitivity, but it remains poorly suited to commodity-level attribution or enforcement decision-making. Biomarkers may be short-lived, non-specific, influenced by non-dietary exposure, or affected by preformed metabolites already present in food. In the proposed framework, biomonitoring should therefore be interpreted as downstream contextual evidence rather than as a primary surveillance or compliance tool.

Future research should prioritise harmonised and regularly updated surveillance datasets, transparent reporting of analytical scope and quantification limits, improved methods for driver-based cumulative risk assessment, and systematic identification and toxicological evaluation of transformation products. Mitigation studies should be designed under realistic fruit-matrix conditions and should report parent-residue reduction together with transformation-product formation, quality outcomes, and commodity-specific feasibility. Broader comparative regulatory work is also needed to include additional jurisdictions and to clarify how global MRL divergence affects enforcement, trade, and risk communication.

## 5. Conclusions

Pesticide residues in fruits should be interpreted through a structured, risk-based framework rather than through detection status or MRL exceedance alone. Evidence from regulated markets indicates high overall compliance, but also confirms that multi-residue occurrence is common enough to require mixture-aware interpretation, particularly when recurrent exposure drivers, high consumers, or sensitive subpopulations are involved.

A decision-oriented pathway links surveillance data with analytical confidence, cumulative risk prioritisation, regulatory contextualisation, and mitigation appraisal. This approach helps distinguish compliance signals from toxicological concern and avoids treating parent-residue reduction as equivalent to hazard elimination when transformation products remain insufficiently characterised. Future work should prioritise harmonised mixture-assessment methods, clearer HRMS and transformation-product reporting, and mitigation studies performed under realistic fruit-matrix conditions.

## Figures and Tables

**Table 1:** Headline compliance metrics from EU and US monitoring programmes.

Programme/ Jurisdiction	Year	Sample Size (n)	No Quantifiable Residues	Residues ≤ Legal Limit	Exceedance of Legal Limit	Non-Compliant	Notes
EU coordinated + national monitoring (EFSA)	2023	132,793	58.0%	38.3%	3.7%	2.0%	EU reporting distinguishes exceedance vs. non-compliance; risk generally assessed as low.
EU monitoring (EFSA)	2022	—	—	96.3% below MRL	—	—	Headline figure: 96.3% of samples below MRL; overall consumer health risk assessed as low.
USDA Pesticide Data Program (PDP)	2022	10,665 (23 commodities)	—	—	—	>99% below EPA benchmarks	Benchmarks are EPA health-based reference levels; compliance metrics do not describe multi-residue complexity.

**Footnote:** The table is not intended as a direct calendar-year comparison between the EU and US programmes. The EU and USDA programmes differ in sampling design, commodity coverage, reporting structure, and risk-screening conventions. The years shown reflect the official datasets used for the corresponding components of this review: EU 2023 for aggregate compliance reporting and USDA PDP 2022 for the commodity-level residue patterns analysed below.

**Table 2:** Dominant residues by commodity (USDA PDP 2022): frequencies and CRA-relevant grouping.

Commodity (PDP 2022)	Dominant Detected Residues (Detection Frequency)	Functional Class	Surveillance-Screening Grouping	Interpretive Note
Blueberries	boscalid 37.3%; azoxystrobin 34.0%; acetamiprid 31.3%; cyprodinil 27.0%	Fungicides + neonicotinoid insecticide	Fungicide cluster (SDHI + QoI + anilinopyrimidine) with secondary neonicotinoid	Multi-residue typical; prioritise fungicides as primary cumulative drivers.
Fresh grapes	fluopyram 49.3%; boscalid 48.0%; cyprodinil 42.9%; tebuconazole 39.2%; fenhexamid 37.4%	Predominantly fungicides	Fungicide cluster (SDHI + anilinopyrimidine + DMI + other)	Highest mixture complexity among listed commodities; fungicides dominate both frequency and likely cumulative contribution.
Peaches	fludioxonil 87.4%; methoxyfenozide 28.2%; spirodiclofen 26.0%	Fungicide + IGR insecticide + acaricide	Single dominant fungicide driver with limited add-ons	Driver-based CRA efficient; assess fludioxonil as primary contributor and add-ons as sensitivity analyses.
Pears	pyrimethanil 63.7%; fludioxonil 50.1%; thiabendazole 45.2%	Fungicides (field + post-harvest)	Anilinopyrimidine + phenylpyrrole + benzimidazole	Mixture reflects both field and post-harvest use; exposure context matters for interpretation.
Plums	fludioxonil 84.5%; methoxyfenozide 28.8% (+ other fungicides)	Fungicide + IGR insecticide	Single dominant fungicide driver with limited add-ons	Similar to peaches: dominant driver simplifies prioritisation.

**Table 3:** Surveillance-derived co-occurrence motifs to guide driver-based cumulative risk assessment (CRA).

Commodity	Co-Occurrence Signal (Pairs ≥5% of Samples)	Dominant Motif	CRA Prioritisation: Primary Drivers	Operational Note
Blueberries	16 pairs ≥5%	Fungicide cluster + neonicotinoid add-on	Model dose addition within fungicide groups; test insecticide contribution separately	Motif supports grouping by mode of action (e.g., SDHI, QoI, anilinopyrimidines) with sensitivity analyses.
Fresh grapes	28 pairs ≥5%	Multi-fungicide cluster	Fungicides as principal drivers; consider sub-grouping (SDHI, DMI, anilinopyrimidine)	Highest complexity; driver-based selection prevents unmanageably large mixture sets.
Peaches	Not reported; dominated by one residue	Single dominant fungicide (fludioxonil) + minor add-ons	Driver-based CRA centred on fludioxonil	Efficient: focus on dominant driver, then incorporate secondary residues if needed.
Pears	Not reported; mixed field + post-harvest pattern	Field/post-harvest fungicide mix	Separate post-harvest vs. field residues before mixture modelling	Avoid collapsing distinct exposure contexts (timing, residue distribution).
Plums	Not reported; dominated by one residue	Single dominant fungicide (fludioxonil) + minor add-ons	Driver-based CRA centred on fludioxonil	As above; dominant driver simplifies mixture assessment.

**Footnote:** In this table, “drivers” refers to surveillance- and exposure-prioritisation signals. It does not imply that all residues within a functional pesticide class share a common toxicological mode of action or should automatically be assigned to the same cumulative assessment group.

**Table 4:** EU/US/Codex divergence examples for citrus residues (EU values audit-supported in Supplement Table S1; US/Codex traceability in Supplement Table S2).

Active Substance	Commodity Basis	EU MRL	US Tolerance	Codex MRL	Primary Source Trail
Imazalil	Oranges (0110020)	4 mg/kg	10 ppm	15 mg/kg	EU 2019/1582; US 40 CFR 180.413; Codex p_id = 110
Fludioxonil	Citrus fruits (0110000)	10 mg/kg	10 ppm	10 mg/kg	EU 2022/1264; US 40 CFR 180.516; Codex p_id = 211
Boscalid	Citrus fruits (0110000)	2 mg/kg	2.0 ppm	2 mg/kg	EU 2021/590; US 40 CFR 180.589; Codex p_id = 221
Thiabendazole	Citrus fruits (0110000)	7 mg/kg	10 ppm	7 mg/kg	EU 2024/1342; US 40 CFR 180.242; Codex p_id = 65
Trifloxystrobin	Citrus fruits (0110000)	0.5 mg/kg	0.6 ppm	0.5 mg/kg	EU 2024/1342; US 40 CFR 180.555; Codex p_id = 213
Thiamethoxam	Citrus fruits (0110000)	0.15 mg/kg	0.40 ppm	0.5 mg/kg	EU 2017/671; US 40 CFR 180.565; Codex p_id = 245
Clothianidin	Citrus fruits (0110000)	0.06 mg/kg	0.07 ppm	0.07 mg/kg	EU 2017/671; US 40 CFR 180.586; Codex p_id = 238
Deltamethrin	Citrus fruits (0110000)	0.02 mg/kg	0.30 ppm (orange)	0.02 mg/kg	EU 2024/1342; US 40 CFR 180.435; Codex p_id = 135
Metalaxyl	Oranges (0110020)	0.7 mg/kg	1.0 ppm	5 mg/kg	EU 2024/1342; US 40 CFR 180.408; Codex p_id = 138

## Data Availability

No new primary datasets were generated in this study. This review was based on publicly available sources, including regulatory surveillance reports, legal and technical documents, and published literature cited in the reference list.

## Supplementary Materials

The following supporting information can be downloaded at: https://www.mdpi.com/article/10.3390/molecules31111980/s1, Supplementary Table S1. EU evidence trail supporting citrus MRL values used in Table 4; Supplementary Table S2. US CFR and Codex traceability record used to populate Table 4; Supplementary Table S3. Mitigation evidence: typical conditions, parent-residue reduction and transformation-product (TP) reporting.

## Abbreviations

The following abbreviations are used in this manuscript:

ADI	Acceptable daily intake
ARfD	Acute reference dose
CAG	Cumulative assessment group
CRA	Cumulative risk assessment
DAPs	Dialkylphosphates
DCCA	3-(2,2-Dichlorovinyl)-2,2-dimethylcyclopropane carboxylic acid
DMI	Demethylation inhibitor
EFSA	European Food Safety Authority
EPA	Environmental Protection Agency
ETU	Ethylenethiourea
EU	European Union
FAO	Food and Agriculture Organization of the United Nations
GAP	Good agricultural practice
GC	Gas chromatography
GC-MS/MS	Gas chromatography–tandem mass spectrometry
GC-Q-Orbitrap	Gas chromatography–quadrupole Orbitrap mass spectrometry
HRMS	High-resolution mass spectrometry
IGR	Insect growth regulator
JMPR	Joint FAO/WHO Meeting on Pesticide Residues
LC	Liquid chromatography
LC-MS/MS	Liquid chromatography–tandem mass spectrometry
LOD	Limit of detection
LOQ	Limit of quantification
MRL	Maximum residue level
MS	Mass spectrometry
MS/MS	Tandem mass spectrometry
NaHCO_3_	Sodium bicarbonate
PDP	Pesticide Data Program
PRBS	Pesticide residue burden score
QoI	Quinone outside inhibitor
QTOF	Quadrupole time-of-flight
SANTE	Directorate-General for Health and Food Safety
SDHI	Succinate dehydrogenase inhibitor
TCPy	3,5,6-Trichloro-2-pyridinol
TMDI	Theoretical maximum daily intake
tMS^2^	Targeted MS squared
TP	Transformation product
US	United States
USDA	United States Department of Agriculture
WHO	World Health Organization
