# Supplementary material for: Pesticide Residues in Fruits: From Surveillance Data to Risk-Based Interpretation and Mitigation
Source: Molecules. 2026 Jun 5;31(11):1980. doi: 10.3390/molecules31111980 (PMC13258465; doi:10.3390/molecules31111980)
Supplement: Supplementary file 1 [file molecules-31-01980-s001.zip › molecules-4311872-supplementary.pdf]

Pesticide residues in fruits: a critical integrative review of surveillance data, cumulative risk drivers, analytical decision-making, regulatory divergence, and mitigation evidence

Supplementary Digital File

CONTENT:

Supplementary Table S1. EU evidence trail supporting citrus MRL values used in Table 4............2

Supplementary Table S2. US CFR and Codex traceability record used to populate Table 4 3

Supplementary Table S3. Mitigation evidence: typical conditions, parent-residue reduction and transformation-product (TP) reporting. 5

Supplementary Table S1. EU evidence trail supporting citrus MRL values used in Table 4

Active substance	EU commodity basis (Annex I code)	EU MRL (mg/kg)	EU legal act (Regulation (EU))
Imazalil	Oranges (0110020)	4	2019/1582
Fludioxonil	Citrus fruits (0110000)	10	2022/1264
Boscalid	Citrus fruits (0110000)	2	2021/590
Thiabendazole	Citrus fruits (0110000)	7	2024/1342
Trifloxystrobin	Citrus fruits (0110000)	0.5	2024/1342
Thiamethoxam	Citrus fruits (0110000)	0.15	2017/671
Clothianidin	Citrus fruits (0110000)	0.06	2017/671
Deltamethrin	Citrus fruits (0110000)	0.02	2024/1342
Metalaxyl	Oranges (0110020)	0.7	2024/1342

Note: MRLs are enforcement limits derived from good agricultural practice and dietary exposure modelling; they are not direct toxicity thresholds. [WHO/FAO, 2009; European Parliament and Council, 2005]

Supplementary Table S2. US CFR and Codex traceability record used to populate Table 4

Active substance	US CFR citation	US tolerance (ppm)	Codex MRL (mg/kg)	Codex record ID	Traceability fields
Imazalil	40 CFR 180.413	10	15	110	Source document; retrieval date; extracted commodity and value.
Fludioxonil	40 CFR 180.516	10	10	211	Source document; retrieval date; extracted commodity and value.
Boscalid	40 CFR 180.589	2.0	2	221	Source document; retrieval date; extracted commodity and value.
Thiabendazole	40 CFR 180.242	10	7	65	Source document; retrieval date; extracted commodity and value.
Trifloxystrobin	40 CFR 180.555	0.6	0.5	213	Source document; retrieval date; extracted commodity and value.
Thiamethoxam	40 CFR 180.565	0.40	0.5	245	Source document; retrieval date; extracted commodity and value.
Clothianidin	40 CFR 180.586	0.07	0.07	238	Source document; retrieval date; extracted commodity and value.
Deltamethrin	40 CFR 180.435	0.30 (orange)	0.02	135	Source document; retrieval date; extracted

					commodity and value.
Metalaxyl	40 CFR 180.408	1.0	5	138	Source document; retrieval date; extracted commodity and value.

Note: US tolerances are commonly expressed in ppm (numerically approximately mg/kg). Cross-jurisdictional differences generally reflect policy and data differences rather than biological differences. [U.S. Environmental Protection Agency, 2024; WHO/FAO, 2009; Codex Alimentarius Commission, 2026]

Supplementary Table S3. Mitigation evidence: typical conditions, parent-residue reduction and transformation-product (TP) reporting.

Method	Typical conditions	Typical parent-residue reduction	Evidence base	TP assessment / reporting	Quality endpoints	Key limitations	Anchor
Running-water washing	Tap water, manual rubbing; minutes-scale	~20–40% (surface residues)	Lab + field; systematic reviews	Rare	Generally preserves texture/organoleptics	Limited for systemic/penetrated residues; variable by wax/surface	Yigit & Velioglu 2020
$NaHCO_3$ wash	1–2% solution; 10–15 min	Up to ~80% for selected surface residues	Controlled laboratory studies	Limited / inconsistent	Minimal in short exposures	Not universal; time- and compound-dependent; penetrated fraction persists	Yang et al. 2017
Peeling	Mechanical removal of peel	Often >80% (surface + some penetrated)	Field-incurred datasets; reviews	Typically not assessed (physical removal)	May reduce peel-associated nutrients/fibre	Not applicable to berries/grapes; trade-off with nutrient loss	Bajwa & Sandhu 2014
Ozone (aqueous / gaseous)	Ozonated water or microbubbles; ~3–18 min	~50–87% (compound- and matrix-dependent)	Lab studies + reviews	Occasional HRMS; not comprehensive	Can affect firmness, colour, aroma at higher doses	TP formation plausible; dose/time optimisation needed	Pandiselvam et al. 2020
Cold plasma (e.g., DBD)	Non-thermal plasma; minutes-scale	Rapid reductions reported (e.g., 47–65% in 4–5 min for anilazine in juice)	Primarily lab / pilot	Limited; research-stage	Potential effects on colour/oxidation depend on setup	TP uncertainty; device- and matrix-specific performance	Ali et al. 2021
Electrolyzed water	Acidic/alkaline; ~5–25 min	Up to ~90% for selected residues	Mixed evidence incl. higher-quality studies	Occasional targeted/HRMS	Often minimal texture impact; depends on pH/time	Protocol heterogeneity; TP hazard seldom evaluated systematically	Studziński et al. 2024
